# Case Report: Pulmonary Alveolar Calcification as a Result of Severe Hypercalcemia due to Acute Lymphoblatic Leukemia.

**DOI:** 10.12688/f1000research.6393.1

**Published:** 2015-05-11

**Authors:** Jose Colleti Junior, Eliana Carla Armelin Benites, Gustavo Spadaccia dos Santos Fernandes, Norberto Antonio Freddi, Walter Koga, Werther Brunow de Carvalho

**Affiliations:** 1 Pediatric Intensive Care Unit, Santa Catarina Hospital , São Paulo, 01310 000, Brazil; 2 Pediatric Oncology Group, Santa Catarina Hospital , São Paulo, 01310 000, Brazil; 3 Radiology Center, Santa Catarina Hospital, São Paulo, 01310 000, Brazil

**Keywords:** hypercalcemia, acute lymphoblastic leukemia, pulmonary alveolar calcification, osteolytic lesions, paediatric case report

## Abstract

Severe hypercalcemia is a rare metabolic disorder in pediatric medicine. This report describes a rare case of severe hypercalcemia and its clinical manifestations in a 2-year-old toddler. The radiological findings caused by hypercalcemia and osteolysis were emblematic of the osteolytic lesions. Hypercalcemia led to massive pulmonary alveolar calcification. The hypercalcemia was successfully treated with pamidronate, a bisphosphonate drug class. Further investigation resulted in a diagnosis of acute lymphoblastic leukemia (ALL). The patient is currently on chemotherapy and has a favorable prognosis. Although severe hypercalcemia alone is an unusual finding as the first sign for ALL, this should be considered, not to mention the radiological images resulted from calcium deposits.

## Introduction

Severe hypercalcemia is unusual in children and can be either caused by elevated parathyroid hormone (PTH) or a PTH-independent mechanism. Primary hyperparathyroidism, familial hypocalciuric hypercalcemia, familial hyperparathyroidism and secondary hyperparathyroidism are examples of PTH-mediated causes of hypercalcemia. In patients with these conditions, in the early stages of the disease, there is a rise in PTH (or inappropriately normal PTH - not suppressed at the beginning of the hypercalcemia), which can confound the differential diagnosis ^1, 2^.

The most common PTH-independent mechanisms for hypercalcemia are related to malignancies ^1– 3^, with many different contributing mechanisms. One such mechanism is local metastatic osteolysis (also known as local osteolytic hypercalcemia or malign humoral hypercalcemia (MHH)), which contributes to hypercalcemia through the production of humoral factors by the tumor. This particular mechanism accounts for 80% of the hypercalcemias related to malignancies ^1, 2^. Most MHH are due to PTHrP and, less frequently, by the production of Vitamin D (1, 25(OH) 2D3). Tumors that secrete PTHrP can induce an increase in bone resorption and calcium reabsorption from the distal kidney tubule, raising the plasmatic calcium from both mechanisms ^1– 3^.

We report a case of alveolar pulmonary calcification after severe hypercalcemia in a 2-year-old toddler with a diagnosis of acute lymphoblastic leukemia (ALL); a rare condition in the pediatric age group. The radiological images of the osteolytic lesions and the alveolar calcification prompt this report, in the hope that they might help to alert clinicians to this unusual condition.

## Case report

A 2-year-old Brazilian white male toddler weighing 11.8kg - previously healthy - presented with vomiting after meals, muscular weakness, generalized pain, abdominal cramps, intestinal constipation and inappetence that had begun 25 days prior to his first examination. There was no relevant information about family history.

He was admitted to the pediatric intensive care unit dehydrated, pallid, referring generalized pain, mainly on legs, arms and in abdomen, and was almost unable to walk. At first, the staff thought it could be sepsis or some endocrinological disorder. However, the initial laboratory tests showed heightened ionic calcium levels in the blood (2.95 mmol/L; normal: 1.11 to 1.40 mmol/L). Other laboratory analyses showed hemoglobin: 9.5 g/mL, white blood cell count: 9,460 cells/mm³, platelet count: 206,000/mm³. Urinalysis showed an elevated leukocyte presence (100 leukocytes/field; normal: < 10/field).

**Figure 1.**
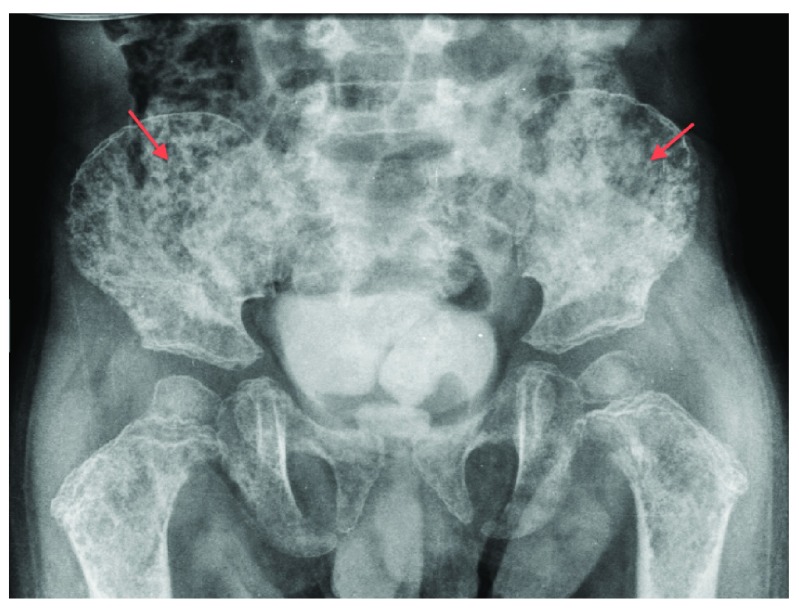
Pelvis X-ray showing osteolytic lesions secondary to bone resorption (red arrows).

As soon as we received the results of the calcium analysis we submitted the patient to a pelvic X-ray to check calcification status. The X-ray revealed substantial osteolysis ( Figure 1).

We started treatment for hypercalcemia with hydration and low doses of furosemide (1mg/kg/day divided into 3 doses, duration 2 days) in order to raise the calcium excretion by the kidneys ^2^. We decided to start pamidronate (0.5mg/kg/day, duration 3 days) – a second-generation bisphosphonate class drug – to stop the osteolysis by the inhibition of calcium resorption ^2^. The symptoms of hypercalcemia subsided and the patient improved.

Subsequent laboratory analysis showed that PTH was low at 8 pg/mL (normal: 10 to 65 pg/mL), calcitonin was normal at 8 pg/mL (normal: less than 12 pg/mL) and 1, 25(OH) 2D3 was low at 21 ng/mL (normal: 30 to 60 ng/mL).

A myelogram was performed and was compatible with acute leukemia. The immunophenotype showed the presence of immature T-type cells that expressed intracytoplasmic CD3 antigens, CD7, CD5, CD1a and partial terminal deoxynucleotidyl transferase (TdT). The presence of CD45 at moderately high levels and a lack of CD2 expression in the studied cells were also observed.

The patient underwent chemotherapy based on the standard Berlin-Frankfurt-Munich (BMF) protocol for pediatric ALL. Complete clinical remission occurred after the first cycle of chemotherapy. As a result, calcium levels returned to normal (ionic calcium: 1.2 mmol/L).

**Figure 2.**
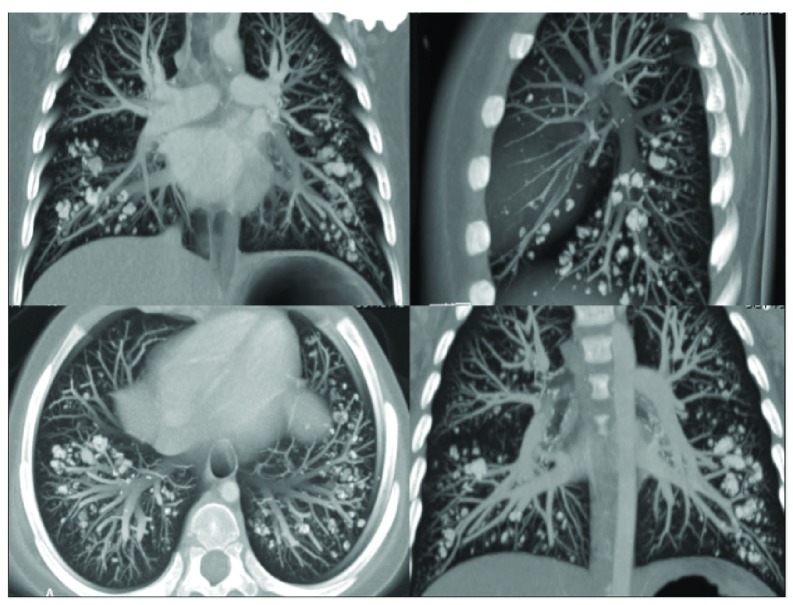
Thorax CT scan: pulmonary alveolar calcification.

Seven months after starting treatment, fever and bacteremia occured, with no associated neutropenia. An infectious disease screen was performed (blood and urine cultures included), and chest x-ray revealed multiple dense nodular structures. A CT scan confirmed structures resembling calcium nodules not exceeding 1 cm in diameter with peribronchovascular distribution, affecting both lungs mainly in the inferior lobes ( Figure 2). Plasma calcium levels (ionized calcium: 1.2 mmol/L) were normal at that time. Tests for fungal infection, and specifically for *Aspergillus spp.*, gave negative results. We also tested for *Cryptococcus neoformans* (agglutination test), *Cytomegalovirus spp.* (antigenemia), tuberculosis (three gastric lavages) and respiratory viruses (nasal secretion tests), with all negative results. The patient had no respiratory symtoms or hypoxemia. Despite the negative results, while the search for an infectious agent was ongoing, he received empiric antibiotic therapy, cefepime (150mg/kg/day) and vancomycin (60mg/kg/day) for 10 days, and lipossomal amphotericin B (5mg/kg/day) for 7 days.

The patient was free from infectious or respiratory disorders. Therefore we attributed the nodules observed in the CT images ( Figure 2) to previous hypercalcemia which possibly led to a process of pulmonary alveolar calcification. We did not perform a biopsy, since we saw no benefit to the patient of doing so.

Presently the patient is on maintenance chemotherapy with methotrexate (20mg/m²/once a week) and 6-mercaptopurine (50mg/m²/day) completing 106 weeks of treatment.

## Discussion

Hypercalcemia in children is rare, especially when it is associated with signs and symptoms that precede a malignant disease. The etiology of hypercalcemia in children is different from adults ^3, 4^. Primary hyperparathyroidism and malignant diseases accounts for 90% of hypercalcemia in adults, but both these conditions are rare in children. The first association between hypercalcemia and malignancy was demonstrated by Myers ^5^. The incidence of hypercalcemia in pediatric malignant diseases has been reported to be from 0.4% to 1.3%.

Alveolar calcification in itself is a rare condition, often associated with hypercalcemia ^6, 7^ and has been reported only rarely in literature ^6, 7^ in association with acute leukemia, making this an unusual finding.

At first, the atypical clinical presentation of this case – the signals and symptoms associated with hypercalcemia - led to a wrong turn in the diagnostic path, seeming to indicate sepsis or endocrine disorders, before we knew about the calcium status. Thereafter, once the cause of the hypercalcemia was detected, our team of specialists acted in harmony and quickly came to a diagnosis.

The treatment of hypercalcemia depends on the primary cause ^3^. Hydration and bone resorption inhibition with bisphosphonate agents are the most important interventions. Bisphosphonate treatment forms the basis of therapy for malignancy-associated-hypercalcemia, and may provide the necessary time for other antitumor therapies to act. Bisphosphonates (in the same mechanism of action used to treat osteoporosis), prevent the bones from losing calcium in primary hyperparathyroidism, but do not decrease the calcium levels. However, when the hypercalcemia is PTH-mediated, surgery is the standard therapy when possible.

The atypical radiological images, if they had not been associated with severe hypercalcemia could have led to unnecessary procedures like biopsies, or the wasteful use of other therapies, like antibiotics and antifungals.

For the clinician it is important to consider a diagnosis of pulmonary calcium alveolar deposits when faced with images resembling what we present in this report, and when other clinical issues are compatible so that a rapid diagnosis is possible, with the hope of sparing the patient unnecessary therapies.

## Consent

Written informed consent for publication of their clinical details and clinical images was obtained from the parent of the patient.
