# Supplementary material for: Case Report: Pulmonary Alveolar Calcification as a Result of Severe Hypercalcemia due to Acute Lymphoblatic Leukemia
Source: F1000Res. 2015 May 11;4:111. [Version 1] doi: 10.12688/f1000research.6393.1 (PMC4505782; doi:10.12688/f1000research.6393.1)
Supplement: Peer review history

# Peer review: Referee response for version 1

**DOI:** 10.5256/f1000research.6859.r9334

**Published:** 2015-07-08

**Authors:** Ana Paula Panzeri Carlotti

**Affiliations:** 1 Division of Pediatric Critical Care, Department of Pediatrics, Ribeirão Preto Medical School, University of São Paulo, Ribeirão Preto, Brazil

The authors report a rare case of severe symptomatic hypercalcemia as the initial manifestation of acute lymphoblastic leukemia associated with massive pulmonary alveolar calcification which was diagnosed several months later. The case is well documented and image findings are striking. This case report may be useful to alert pediatricians for the diagnosis of malignancy and also favor an early investigation of metastatic calcification of soft tissues in patients with such a severe degree of hypercalcemia.

I have read this submission. I believe that I have an appropriate level of expertise to confirm that it is of an acceptable scientific standard.

# Peer review: Referee response for version 1

**DOI:** 10.5256/f1000research.6859.r8865

**Published:** 2015-06-02

**Authors:** Jerry Zimmerman

**Affiliations:** 1 Center for Clinical and Translational Research, Seatlle Children's Hospital, Seattle, WA, USA

This article submitted by Colleti and colleagues from Santa Catarina Hospital, São Paulo, Brazil, provides an interesting case report of an unusual entity, namely pulmonary alveolar calcification. The report is well documented and the conclusions not overstated. However, additional data, if available would be of interest.  Specific comments regarding the manuscript are noted below:

1. ‘Acute lymphoblastic leukemia’, not ‘acute lymphoblatic leukemia’.
2. At the time of initial presentation, what was the calcium x phosphate product? If the tumor burden was high and ongoing lysis of leukemia cells was occurring, serum phosphate might also be expected to be elevated and contributing to the calcification of soft tissue.
3. Did the patient present with hypertension? 
4. Was a shortened QT interval noted on the admission EKG?
5. Did the medical team specifically inquire about a family history of multiple endocrine neoplasia?
6. At the time of initial presentation, did radiographs demonstrate any evidence of soft tissue calcification? Was calcification of the lungs present on admission; was nephrolithiasis present?
7. The CT scan reproduced in Figure 2, also appears to demonstrate calcification of the heart and great vessels, as well as severe osteopenia of the lower thoracic vertebrae. Did the patient exhibit any structural cardiac problems, such as calcified valve leaflets by echocardiography? Did the patient exhibit any neurological findings attributable to thoracic vertebral osteolyis?
8. Although biphosphonates represent the safest approach to therapy for this patient, is there any role for calcitonin, mithramycin, and indomethacin?
9. It is probably worth mentioning pulmonary alveolar microlithiasis as well as the SLC34A2 gene mutation in the Na/PO _4 _co-transporter, as other causes of pulmonary alveolar calcification.
10. Given the appearance of thoracic CT imaging, longitudinal pulmonary function testing is probably warranted for the patient presented.

I have read this submission. I believe that I have an appropriate level of expertise to confirm that it is of an acceptable scientific standard.

# Response

**Published:** 2015-06-21

**Authors:** Jose Colleti Junior

**Affiliations:** Hospital Santa Catarina, Brazil

The comments of Dr Zimmerman about this article are relevant and helpful.

I will try to elucidate some questions as follows:

1. The phosphate was really high at the initial presentation, contributing to the calcification of soft tissue;
2. The patient did not have hypertension;
3. The initial EKG was normal (we did not expect that);
4. There was no evidence of family neoplasias;
5. We considered other drugs, besides bisphosphonates, to treat the hypercalcemia. We decided on pamidronate because of the availability, and our personal experience;
6. Although the thoracic CT imaging is striking, the patient did not present any clinical signal of respiratory disorder.
7. The patient is still undergoing treatment and is doing well.
